# Topical Meloxicam Hydroxypropyl Guar Hydrogels Based on Low-Substituted Hydroxypropyl Cellulose Solid Dispersions

**DOI:** 10.3390/gels10030207

**Published:** 2024-03-18

**Authors:** Zaid Dahma, Carlos Torrado-Salmerón, Covadonga Álvarez-Álvarez, Víctor Guarnizo-Herrero, Borja Martínez-Alonso, Guillermo Torrado, Santiago Torrado-Santiago, Paloma Marina de la Torre-Iglesias

**Affiliations:** 1 Department of Pharmaceutics and Food Technology, Faculty of Pharmacy, Complutense University of Madrid, Plaza Ramón y Cajal s/n, 28040 Madrid, Spain; zdahma@ucm.es (Z.D.); ctorrado@ucm.es (C.T.-S.); covadong@ucm.es (C.Á.-Á.); 2 Instituto Universitario de Farmacia Industrial, Complutense University of Madrid, Plaza Ramón y Cajal s/n, 28040 Madrid, Spain; 3 Department of Biomedical Science, Faculty of Pharmacy, University of Alcalá de Henares, Ctra Madrid-Barcelona Km 33600, 28805 Madrid, Spain; victor.guarnizo@uah.es (V.G.-H.); borja.martineza@uah.es (B.M.-A.); guillermo.torrado@uah.es (G.T.)

**Keywords:** meloxicam, low-substituted hydroxypropyl cellulose, hydroxypropyl guar, solid dispersion, hydrogels, polymer/polymer interactions

## Abstract

Meloxicam (MX) is a poorly water-soluble drug with severe gastrointestinal side effects. Topical hydrogel of hydroxypropyl guar (HPG) was formulated using a solid dispersion (SD) of MX with hydroxypropyl cellulose (LHPC) as an alternative to oral administration. The development of a solid dispersion with an adequate MX:LHPC ratio could increase the topical delivery of meloxicam. Solid dispersions showed high MX solubility values and were related to an increase in hydrophilicity. The drug/polymer and polymer/polymer interactions of solid dispersions within the HPG hydrogels were evaluated by SEM, DSC, FTIR, and viscosity studies. A porous structure was observed in the solid dispersion hydrogel MX:LHPC (1:2.5) and its higher viscosity was related to a high increase in hydrogen bonds among the –OH groups from LHPC and HPG with water molecules. In vitro drug release studies showed increases of 3.20 and 3.97-fold for hydrogels with MX:LHPC ratios of (1:1) and (1:2.5), respectively, at 2 h compared to hydrogel with pure MX. Finally, a fitting transition from zero to first-order model was observed for these hydrogels containing solid dispersions, while the *n* value of Korsmeyer–Peppas model indicated that release mechanism is governed by diffusion through an important relaxation of the polymer.

## 1. Introduction

Meloxicam (MX) is a powerful nonsteroidal anti-inflammatory drug (NSAID) used to treat inflammation and pain associated with arthritis, osteoarthritis, and rheumatic diseases [1,2]. Due to the recurrent gastrointestinal complications related to oral administration of MX, many alternative routes of administration become necessary. The topical administration method provides several advantages over oral administration: the selective capacity to deliver the active ingredient to a specific site, avoid the first-pass effect, decrease gastrointestinal side effects, and improve patient compliance [3]. For these reasons, topical administration of MX or other NSAIDs is recommended, before the oral route, for the treatment of inflammation, edema, and mild to moderate osteoarthritis pain [4,5]. MX is a drug with negligible aqueous solubility [1]. Possibly, this characteristic influences the lack of available marketed formulations with MX hydrogels. Therefore, improvements in solubility and dissolution are relevant to increase the transport of MX into the skin [4,6,7]. Regarding hydrogel’s formulations, characteristics such as hydrosolubility, viscosity, and polymer chain length could change the drug release [8,9,10]. Natural or semi-synthetic polymers such as alginate, xanthan gum, and hydroxypropylmethyl cellulose [3,6,10] or synthetic polymers such as lauroyl polyoxyl-32 glycerides (Gelucire^®^ 44/14), polyvinyl alcohol/polyvinyl pyrrolidone (PVA/PVP), or polyoxyethylene polymers (Poloxamer 407) [3,5,9,11] have been used to prepare fast dissolving hydrogels.

Solid dispersions (SDs) are widely used to enhance the dissolution of poorly soluble drugs. The main classification criterion of polymeric carriers is their physical state within the solid dispersion. Polymeric carriers can be in a disordered amorphous state with fast solubility and dissolution or in an ordered crystalline state, which is usually related to the limited solubility of the drug [8]. Solid crystalline dispersions with polymers such as polyethylene glycol or poly (methylvinyl ether-co-maleic acid) were used in topical hydrogel [1,11]. Amorphous SDs with different hydrophilic polysaccharides such as polyvinyl pyrrolidone and Eudragit RSPO were employed to improve the dissolution of poorly soluble drugs from topical hydrogels [3,5].

In the study of solid dispersions, different parameters were used to increase the solubility of poorly soluble drugs, such as the hydrophilic characteristics of the carrier [3,10] and the decrease in crystallinity, determined by DSC and XRPD studies [5,7]. These parameters will be evaluated for selecting the most appropriate drug:carrier ratios to increase the delivery of poorly soluble drugs. In recent years, amorphous polymers such as hydroxypropyl methyl cellulose, hydroxypropyl cellulose, and croscarmellose have been used to improve the solubility of poor drugs [12,13]. Several previous works showed different amorphous solid dispersions with low-substituted hydroxypropyl cellulose (LHPC) as a hydrophilic carrier. The high swelling capacity of LHPC in the dissolution medium increases the solubility of poorly soluble drugs onto the skin surface [14,15]. These excipients have an amphiphilic nature that improves the absorption of drug molecules within the polymer chains and decreases the thermodynamic instability of amorphous forms [5]. Differential scanning calorimetry (DSC) and X-ray powder diffraction (XRPD) studies are used to study the decrease in crystallinity of drugs such as MX with different ratios of MX: hydrophilic polymers [5,15]. Therefore, the thermodynamic activity of the drug that can be achieved in this polymer can increase its concentration within the hydrogel and improve its skin permeability [3].

To study drug release under conditions similar to those observed on the skin surface, the different SD hydrogel formulations are evaluated with water or acetate buffer pH 5.5. These media have been commonly used in dissolution studies with different hydrogels for topical administration [14,16]. Furthermore, the simulated medium at pH 5.5 proved to be suitable for showing significant differences in solid dispersions of poorly soluble drugs [16]. Finally, the different kinetic studies of topical hydrogels are adequate to relate the dissolution profiles with the degree of drug/polymer interaction observed for the different SDs [2,17,18].

The aim of this work is to prepare hydrogels with MX solid dispersions that allow a fast release of MX topically. The solid dispersions will be based on LHPC, as a hydrophilic carrier, studying the influence of different proportions of MX:LHPC. The solubility studies will allow us to select the MX:LHPC ratios that can be used in the preparation of the MX solid dispersions. Fourier-transform infrared spectroscopy (FTIR), X-ray powder diffraction (XRPD), and differential scanning calorimetry (DSC) techniques were employed to study the drug/polymer and polymer/polymer interactions with the different amorphous solid dispersions of MX (MX − ASD). The dissolution study in a simulated skin medium (acetate buffer pH 5.8) will be suitable to evaluate the improvements in the release of MX ASD hydrogels with different ratios of MX:LHPC. The MX transport mechanism for the different MX ASDs in hydrogel formulations was examined by fitting experimental data to several model equations and calculating the related parameters.

## 2. Results and Discussion

### 2.1. Solubility Studies at pH 5.8

**Figure 1:**
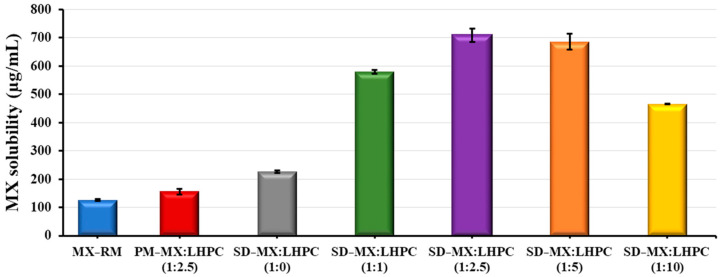
Solubility degree in phosphate buffer (pH 5.8) of MX raw material (MX − RM), physical mixture PM − MX:LHPC (1:2.5) and solid dispersions: SD − MX:LHPC (1:0), SD − MX:LHPC (1:1), SD − M:LHPC (1:2.5), SD − MX:LHPC (1:5), and SD − MX:LHPC (1:10).

The aim of this study is to assess how varying SD − MX:LHPC ratios affect the solubility characteristics of the drug, which could improve its dissolution performance, thereby impacting its topical delivery [19]. In Figure 1, it is shown the solubility of the MX raw material (MX − RM) in acetate buffer pH 5.8 is 125.76 ± 2.93 µg/mL. However, a slight increase was observed for the physical mixture PM − MX:LHPC (1:2.5) with solubility values of 156.36 ± 2.20 µg/mL. This slight increase in solubility values was attributed to the hydrophilic character of the LHPC chains.

The drug solubility coefficient has slightly improved in the solid dispersion SD − MX: LHPC − 1:0 (226.61 ± 3.78 µg/mL) which was probably due to the vacuum drying process and the SDS addition [20].

Nonetheless, solid dispersions containing LHPC exhibited a notable enhancement (*p* < 0.05) in solubility, demonstrating 4.61, 5.64, and 5.45-fold increases for SD − MX:LHPC − 1:1, SD − MX:LHPC − 1:2.5, and SD − MX:LHPC − 1:5, respectively, when compared to pure MX. LHPC seems to help formulate moisturize, achieving higher dissolution values [15]. However, the solid dispersion SD − MX:LHPC (1:10) showed a significant decrease (*p* > 0.05) in its solubility value (465.69 ± 2.13 µg/mL) compared to SD − MX:LHPC (1:2.5). This result could be attributed to an increased LHPC content, which might hinder the mobilization and solubility of MX. Comparable decreases in solubility values have been noted with elevated proportions of other hydrophilic polymers [13]. Previous studies with hydrophilic polymers have demonstrated the suitability of employing low carrier ratios in solid dispersions [21].

Solid dispersion SD − MX:LHPC − 1:2.5 exhibited the highest solubility coefficient (709.17 ± 24.02 µg/mL) among LHPC formulations. Probably, this ratio of SD − MX:LHPC (1:2.5) forms a more porose structure, improving water absorbance [22]. This could be attributed to the freeze-drying process, which favors the entry of MX molecules into the interpenetration LHPC network [23] and in the conversion of the drug’s crystalline form to an amorphous form [24]. These results allow us to select the following SDs:SD − MX:LHPC (1:1), SD − MX:LHPC (1:2.5), and SD − MX:LHPC (1:5) for further characterization and dissolution studies.

### 2.2. DSC Studies

**Figure 2:**
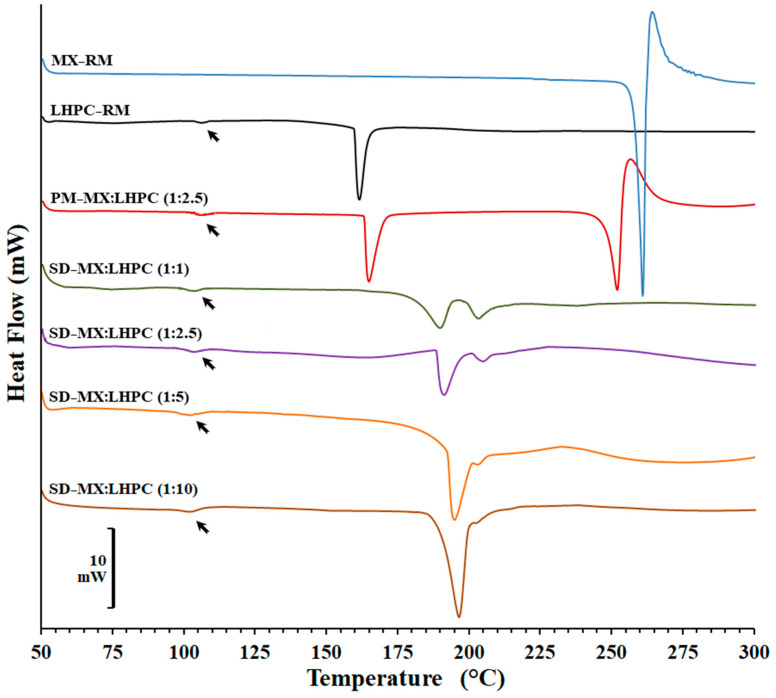
DSC thermograms of MX and LHPC raw materials (MX − RM and LHPC − RM), physical mixture PM − MX:LHPC (1:2.5) and solid dispersions: SD − MX:LHPC (1:1), SD − M:LHPC (1:2.5), SD − MX:LHPC (1:5), and SD − MX:LHPC (1:10).

Figure 2 displays the DSC scans for MX and LHPC raw materials, as well as the physical mixture PM − MX:LHPC (1:2.5) and various solid dispersions: SD − MX:LHPC (1:1), SD − MX:LHPC (1:2.5), SD − MX:LHPC (1:5), and SD − MX:LHPC (1:10).

The DSC scan for meloxicam raw material (MX − RM) exhibited a sharp endotherm at 261.77 °C with an enthalpy of fusion of −84.51 J/g, followed by an exothermic peak, suggesting that the substance melts with decomposition. This behavior is typical of a regular orthorhombic microcrystalline form [25,26]. The thermogram of the carrier (LHPC) showed a slight endothermic/exothermic transition at 106.72 °C and a melting peak at 166.68 °C (enthalpy value of −52.14 J/g) characteristic of a semi-crystalline structure [14,27]. A comparable glass transition of LHPC at similar temperatures has been previously described in DSC studies [14,15].

The physical blend PM − MX:LHPC (1:2.5) displayed a slight glass transition at 107.32 °C characteristic of the LHPC polymer, and two broad endothermic peaks at 169.69 °C and 252.19 °C corresponding to LHPC and MX, respectively. The shift to the left and the reduction in enthalpy values could be attributed to a slight polymorphic transition that occurred during the mixing MX and a dilution effect of the drug within the carrier [26,27,28]. In addition, the substantial temperature disparity between the two endothermic peaks suggests strong compatibility between the MX and LHPC polymeric chains [29].

The SD thermograms for SD − MX:LHPC (1:1), SD − MX:LHPC (1:2.5), SD − MX:LHPC (1:5), and SD − MX:LHPC (1:10) showed a slight endothermic/exothermic transition between 107.12–110.07 °C and a first endothermic peak (between 190.51–195.32 °C), which were related to the semicrystalline structure of LHPC [14,15]. Furthermore, in the MX solid dispersions, a positive interaction between LHPC and MX could be recognized, which is responsible for the shift of the carrier melting peak towards higher temperatures. Whereas all SDs showed important decreases in the second endothermic peak (between 207.32–202.12 °C) attributed to MX. With an increase in the proportion of LHPC in the SDs, the melting peaks of MX became broader and shifted further toward a lower temperature. The significant decreases in the MX crystallinity percentages (18.12%, 8.62%, 4.30%, and 4.10%, respectively). The low crystallinity of SD − MX:LHPC (1:2.5) could be related to the entrance of drug molecules within the LHPC polymeric network. However, the higher ratio of LHPC in SD − MX:LHPC (1:5) and SD − MX:LHPC (1:10) does not greatly enhance the amorphous forms of MX. The presence of partial crystalline forms in solid dispersions with high ratios of semi-crystalline carriers has been previously described in different studies [3,14].

The low crystallinity of SD − MX:LHPC (1:2.5) could be related to the entrance of drug molecules within the LHPC polymeric network. The conversion from crystalline to amorphous states of poorly soluble drugs within a semicrystalline structure with different cellulose polymers has been previously confirmed [12,13].

### 2.3. X-ray Powder Diffractometry (XRPD)

XRPD analyses were conducted to investigate the changes in crystallinity after the freeze-drying process, as well as the effect of adding different ratios of LHPC carrier to the solid dispersion.

**Figure 3:**
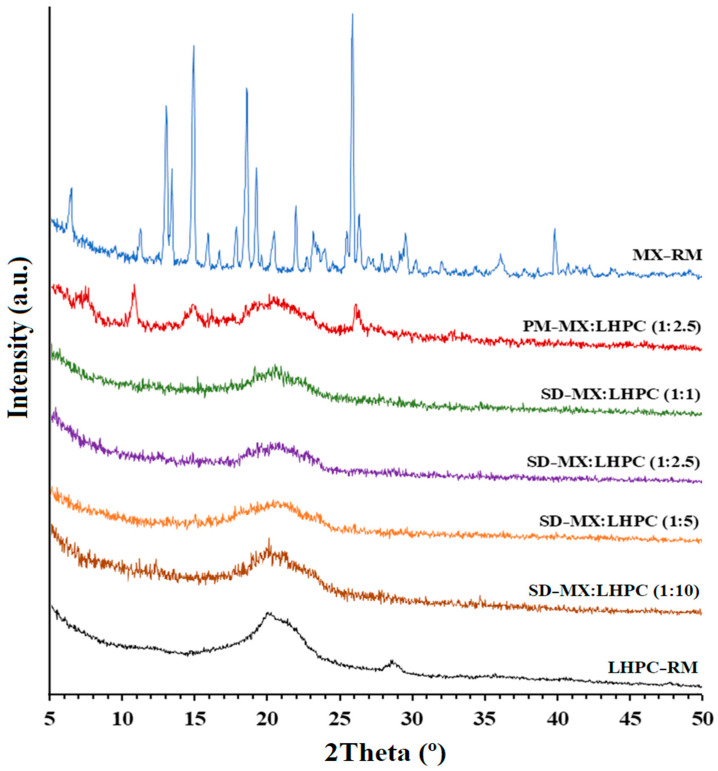
X-ray powder diffraction scans of MX and LHPC raw materials (MX − RM and LHPC − RM), physical mixture PM − MX:LHPC (1:2.5) and solid dispersions: SD − MX: LHPC (1:1), SD − M:LHPC (1:2.5), SD − MX:LHPC (1:5), and SD − MX:LHPC (1:10).

In Figure 3, the X-ray diffraction profiles of MX and LHPC raw materials (MX − RM and LHPC), physical mixture PM − M:LHPC (1:2.5) and the solid dispersions: SD − MX:LHPC (1:1); SD − MX:LHPC (1:2.5); SD − MX:LHPC (1:5), and SD − MX:LHPC (1:10) are depicted.

The crystal structure of the pure drug, MX − RM, exhibited representative low-intensity peaks at angles of 13.1°, 13.4°, 14.9°, 18.6°, 19.2°, and 25.8° (2θ), as reported by other authors [30,31]. The low-intensity values observed in its diffraction peaks are characteristic of a drug substance with low crystallinity. The XRPD profile of LHPC displayed a distinctive semi-crystalline halo diffraction pattern ranging from 15.7° to 25.1° (2θ), with a majority diffraction intensity at 20.1° (2θ) [32]. The physical blend PM − MX:LHPC (1:2.5) exhibited the distinctive peaks of MX − RM and a broad semi-crystalline halo spanning from 15.7° to 24.8° 2θ, indicative of the LHPC polymer (Figure 3). The reduction in the intensity of MX peaks in PM − M:LHPC (1:2.5) could be due to a slight polymorphic transition that occurred during the mixing of MX and a dilution effect [28].

The SD with the lower amount of carrier, SD − MX:LHPC (1:1) displayed a small peak at 25.8° (2θ), which was related to the representative peak of maximum intensity for the MX − RM. The decreased crystallinity for MX within SD − MX:LHPC (1:1) was attributed to the presence of LHPC carriers during the freeze-drying process. Similar crystallinity decreases have been observed in solid dispersions with low proportions of hydrophilic carriers. This important decrease in crystallinity could be related to significant improvements in the dissolution profiles [29].

However, SD − MX:LHPC (1:2.5), SD − MX:LHPC (1:5), and SD − MX:LHPC (1:10) did not show any of the peaks attributed to MX. This result supports that with these LHPC ratios, the MX molecules were included within the LHPC polymeric matrix. Nevertheless, the greater intensity of the semicrystalline halo between 15.7–25.1° (2θ) observed in SD − MX:LHPC (1:10) was related to the greater amount of the semicrystalline polymer LHPC. These results indicate that SD − MX:LHPC (1:10) exhibited a dense structure that could delay the absorption of water within the interpenetration network, resulting in decreased MX solubility. This outcome validates the inclusion of MX within the LHPC polymeric matrix, consistent with observations from the DSC studies. In this context, the interactions between the drug and the LHPC polymer lead to a marked reduction in MX crystallinity, potentially facilitating rapid drug dissolution due to its elevated Gibbs free energy [33].

### 2.4. FTIR Spectroscopy Study

**Figure 4:**
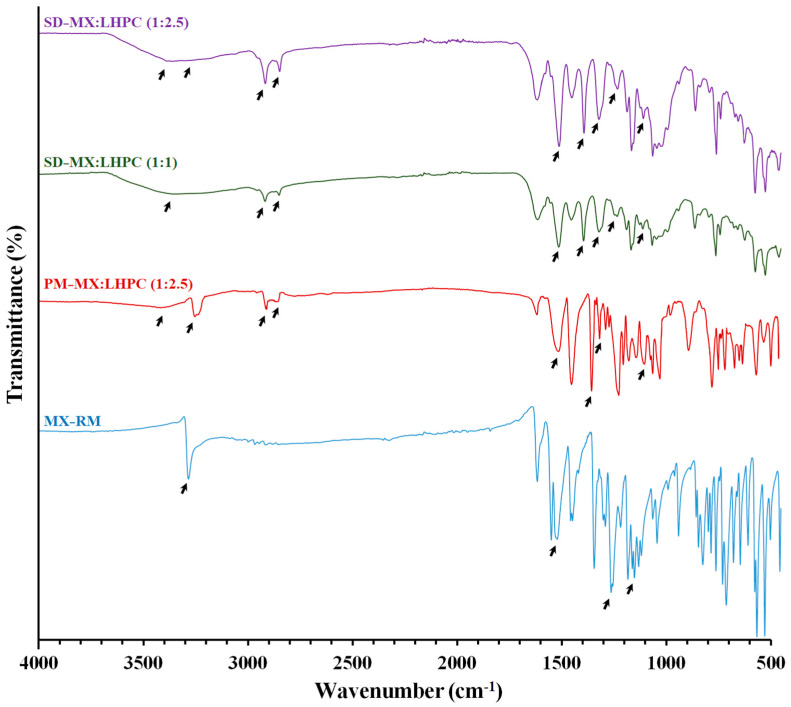
FTIR spectra of MX raw material (MX − RM), physical mixture PM − MX:LHPC (1:2.5), and solid dispersions: SD − MX: LHPC (1:1) and SD − M:LHPC (1:2.5).

The FTIR spectra shown in Figure 4 provide useful information about the functional groups and potential drug/carrier interactions in the physical mixture and SDs. The spectrum of MX raw material (MX − RM) showed the following characteristic bands: at 3285 cm^−1^, matching to N–H stretching vibrations of the secondary amide; at 1520 cm^−1^, attributed to C=N stretching vibrations of the thiazole; a band at 1262 cm^−1^, related to C–N stretching vibrations of the amine; and a band at 1161 cm^−1^ (C–O–C stretching vibrations) [34]. The presence of MX in the physical mixture PM − MX:LHPC (1:2.5) showed characteristic vibration peaks at 3285, 1513, 1262, and 1157 cm^−1^. The peaks at 3381, 2918, 2890, and 1619 cm^−1^, were characteristic of –OH, C–H, and benzene ring skeleton, and the band at 1161 cm^−1^ was related to C–O–C stretching vibrations. In addition, the peaks at 1394 and 1319 cm^−1^ were attributed to –OH and C–O stretching vibrations for the LHPC polymer [35]. The absence of changes in the main frequencies of MX and LHPC for the physical mixture spectrum indicated the lack of MX–polymer interaction [34,36]. However, both solid dispersions showed a slight shift for the vibration peaks at 3383 and 3389 cm^−1^, which were related to –OH stretching bands for SD − MX:LHPC (1:1) and SD − MX:LHPC (1:2.5), respectively. While the vibration peaks were shifted to 1394 and 1395 cm^−1^ for the –OH stretching bands, and also at 1320 and 1323 cm^−1^ for the C–O stretching bands for SD − MX:LPC (1:1) and SD − MX:LHPC (1:2.5), respectively. Therefore, these changes detected in the solid dispersions SD − MX:LHPC (1:1) and SD − MX:LHPC (1:2.5) could be attributed to the hydrogen bonding interaction. This interaction was produced among the hydroxyl moieties from MX and the –OH and C–O moieties from LHPC [36]. The following bands at 3285, 2909, 2893, and 1520 cm^−1^, related to N–H, C–H, and C=N stretching vibrations from LHPC polymer and MX, did not change. While the C–N and C–O–C bands (1262 and 1118 cm^−1^) of MX were overlapped by the bands of the LHPC [17], suggesting that the drug was also dispersed into the LHPC carrier [14]. The hydrogen bonding interaction described in SD − MX:LHPC (1:2.5) was related to a significant improvement in dissolution studies [14,17].

### 2.5. Characterization of HPG Hydrogels

#### 2.5.1. Viscosity Studies

Previously, a viscosity study for HPG blank hydrogel (H-Blank) at concentrations of 1.0 to 2.5% (*w*/*v*) was carried out in order to develop topical hydrogels with good characteristics for their administration on the skin. The viscosity results for these hydrogels with a constant shear rate of 50 s^−1^ were 9.22 ± 0.26, 12.18 ± 0.26, and 17.45 ± 0.28 Pa·s for blank HPG hydrogels with percentages of 1.0, 1.75 and 2.5%, respectively. These results showed that a gelling agent concentration of 1.75% (*w*/*v*) HPG produced a gel with adequate viscosity values, which would be suitable for topical administration [5,37]. This concentration was selected for further analysis: viscosity, FTIR, SEM, dissolution, and kinetic studies.

**Figure 5:**
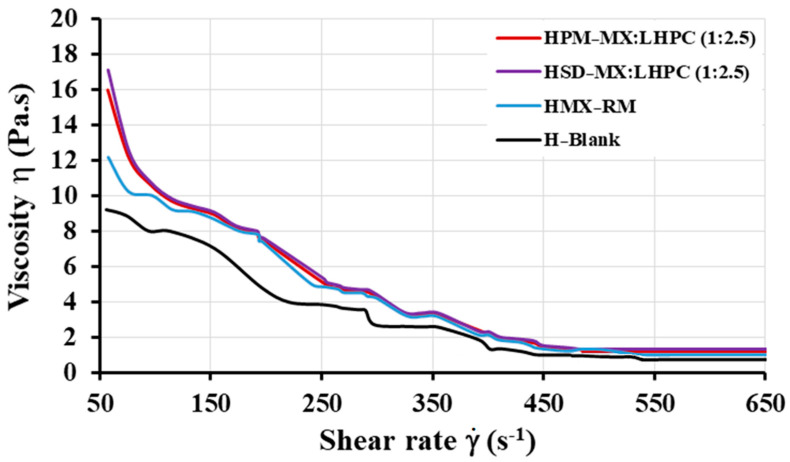
Rheological behavior of the hydrogel formulations: H − Blank, HMX − RM, HPM − MX:LHPC (1:2.5), and HSD − MX:LHPC (1:2.5). Results are presented as mean values (*n* = 3) for each formulation.

The viscosity of all hydrogels: H-Blank, MX raw material hydrogel (HMX − RM), physical mixture hydrogel HPM − MX: LHPC (1:2.5) and solid dispersion hydrogel HSD − MX: LHPC (1:2.5) decreased when the shear rate increased (Figure 5), this behavior of the HPG hydrogels favors the flow and fluidity of the topical formulations [5]. Furthermore, under a constant rate cycle of 50 s^−1^, the mean viscosity values at 25 °C were 12.18 ± 0.26 Pa·s for H-Blank and 12.46 ± 0.42 Pa·s for HMX − RM, respectively. Similar increases at low shear rate values (50 s^−1^) have been previously observed in guar gum hydrogels loaded with different drugs, maintaining a decreased viscosity when the shear rate increases, which is a critical property for easy topical administration [37]. In addition, different studies indicated that the viscosity of the hydrogels played an important role in the percentage of drug release [6].

Viscosity studies for HPM − MX:LHPC (1:2.5) showed that the incorporation of both MX and LHPC to the HPG hydrogel significantly increases (*p* > 0.05) the viscosity (15.99 ± 0.80 Pa·s at 50 s^−1^) compared to H-Blank. These high viscosity values could be due to two different factors: the first one is the presence of a dispersed phase with the HPG gel [38]; and the second one is the increased number of hydrogen bonds that can be formed between hydroxyl groups in adjacent molecules, resulting in MX/LHPC and LHPC/HPG interactions observed in the FTIR studies [35].

Finally, the maximum increase in viscosity values (17.12 ± 0.96 Pa·s at 50 s^−1^) was seen for HSD − MX:LHPC (1:2.5) compared to HPM − MX:LHPC (1:2.5) [39]. Possibly the interactions between LHPC and HPG produce an increase in free hydroxyl groups on the network surface, able to produce polymer/water interactions observed in the FTIR spectra. This greater interaction by hydrogen bonds could explain the increase in the viscosity of the hydrogel in the solid dispersion [5].

In general, the viscosity values for all hydrogel formulations at 50 s^−1^ and the decrease in their viscosity when the shear rate increased indicated suitable characteristics to facilitate the extraction from the packaging material and ensured improved spreadability in the affected area, thus improving the topical delivery of sparingly soluble drugs.

#### 2.5.2. Scanning Electron Microscopy (SEM)

**Figure 6:**
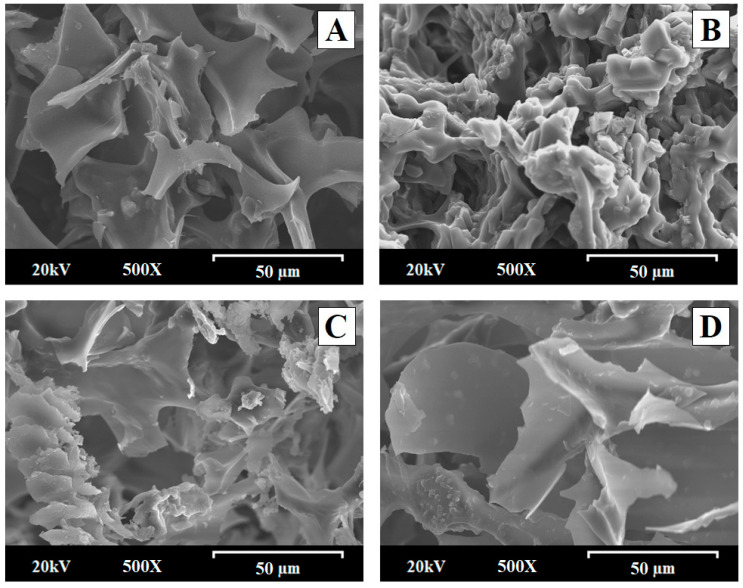
Scanning electron micrographs of freeze-dried formulations: (**A**) HPG hydrogel (H − Blank); (**B**) MX hydrogel (HMX − RM); (**C**) physical mixture hydrogel HPM − MX:LHPC (1:2.5); (**D**) solid dispersion hydrogel HSD − MX:LHPC: (1:2.5). Original magnification is 500× and the scale bar is equal to 50 μm.

Figure 6 shows the morphology observed by scanning electron microscopy of the freeze-dried hydrogels: HPG hydrogel (H-Blank), meloxicam hydrogel (HMX − RM), physical mixture hydrogel HPM MX:LHPC (1:2.5), and solid dispersion hydrogel HSD − MX:LHPC (1:2.5). These formulations were taken at a high magnification of 500×.

The freeze-dried H-Blank exhibited a sleek appearance, with prominent pores visible on the surface. The selected percentage of hydroxypropyl guar gum (1.75% *w*/*w*) produced an interpolymer structure with highly porous structures (50–80 µm) that establish connections (channels) within the interior of the system. Analogous highly porous formations have been previously reported for different freeze-dried topical hydrogels. These structures are characteristic of SEM studies of hydrogels formed by the polymer relaxation in an aqueous medium after a freeze-drying process [16,40]. Scanning electron micrographs of meloxicam HMX − RM hydrogel (Figure 6) exhibited a characteristic morphology of crystalline aggregates with sizes of around 10–20 µm and a smooth surface [41]. The physical hydrogel mixture HPM − MX: LHPC: (1:2.5), showed a dense porous structure with smaller interconnected pores around 40–60 µm attributed to the presence of LHPC within the freeze-dried hydrogel. In this physical mixture, it is possible to observe the crystalline aggregates attributed to MX. These MX’s crystal groups presented sizes around 10–20 µm similar to MX − RM. Previous studies revealed that the addition of hydrophilic polymers in the hydrogel altered its porous structure [5]. The solid dispersion hydrogel HSD − MX:LHPC (1:2.5) showed the largest changes in the structure of the freeze-dried hydrogel. The high hydration capacity of the solid dispersion presented a decrease in the pore size of the hydrogels [4,16]. HSD − MX: LHPC: (1:2.5) displayed an absence of MX’s crystal groups, and it is only observed the presence of small crystals (1–5 µm) within the LHPC fibers (Figure 6). Similar crystal inclusion processes have been previously described in solid dispersion employing other cellulose derivatives as carriers [13].

#### 2.5.3. Fourier Transform Infrared Spectroscopy for the Different Hydrogels

**Figure 7:**
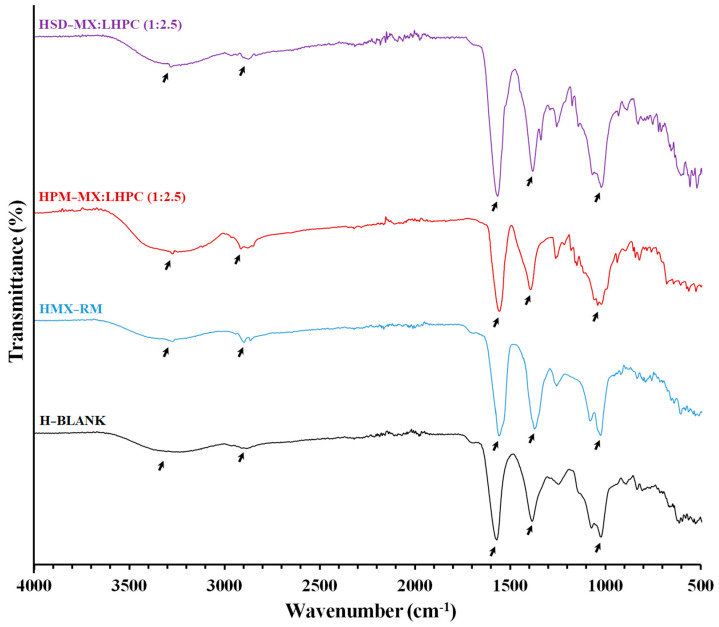
FTIR spectra of HPG hydrogel (H − Blank), MX hydrogel (HMX − RM), physical mixture hydrogel HPM − MX:LHPC (1:2.5), and solid dispersion hydrogel HSD − MX: LHPC (1:2.5).

FTIR analyses of freeze-dried hydrogels, shown in Figure 7, were conducted for the evaluation of possible drug/polymer, and polymer/water molecular interactions. The freeze-dried H-Blank hydrogel exhibited a spectrum with bands at 3236 cm^−1^, related to –OH stretching representative of HPG polymer/water interaction. The water molecules were also evidenced by –OH broad deformation bands at about 1575 and 1389 cm^−1^ resulting from bending vibrations [25,35]. Both bands at 2900 and 1027 cm^−1^ were attributed to C–H and C–O–H stretching vibrations, respectively, characteristic of the HPG polymer [17,40]. Furthermore, this formulation displayed a higher intensity for the bands related to the –OH stretching vibration from the hydroxylic groups and the band of C–O–H, which were involved with hydrogen bonding between the polymeric chains and the aqueous medium [25]. The HMX − RM and HPM − MX:LHPC (1:2.5) hydrogels showed a characteristic peak at 2904 representative of C–H stretching vibrations, and the bands at 3239, 1575, 1391, and 1029 cm^−1^ were related to –OH and C–O–H deformations caused by the interaction between HPG/water, all of them characteristic of freeze-dried HPG hydrogel [25,35]. The bands at 3236 and 1263 cm^−1^ were related to the N–H and C–N stretching vibrations, respectively, from the MX [34]. The absence of other MX bands was attributed to overlapping bands of the HPG hydrogel. In addition, the lack of changes in the HMX − RM and HPM − MX:LHPC (1:2.5) FTIR spectra indicated that there was no interaction between the MX and HPG polymer [16,17]. Finally, HSD − MX:LPC (1:2.5) hydrogel showed a peak at 3289 cm^−1^ attributed to the overlap of the N–H band within the –OH band for the HPG hydrogel. Moreover, the band at 2904 cm^−1^ did not change and was related to C–H stretching vibrations. Nevertheless, peaks at 1579, 1391, and 1032 cm^−1^ representative of the hydroxyl bands and C–O–H stretching vibrations of the freeze-dried HPG hydrogel, were slightly shifted to higher wavenumbers [40]. The blue shift may result from the hydrogen bonding interactions between the hydroxyl groups of LHPC and HPG polymeric chains, along with water molecules [35].

#### 2.5.4. In Vitro Release Profile Study

**Figure 8:**
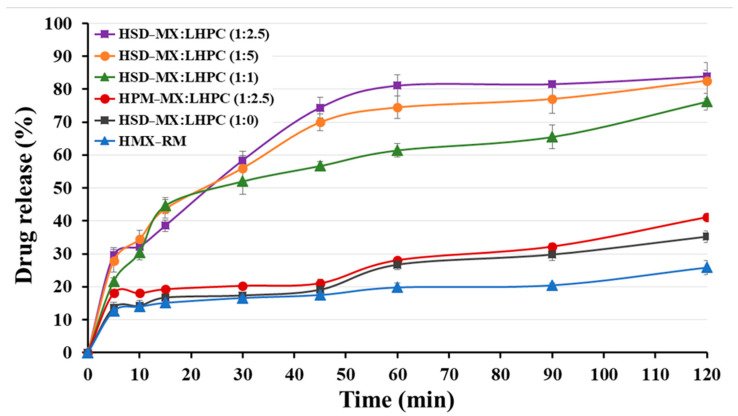
Release profiles at pH 5.8 for meloxicam hydrogels: HMX − RM, HPM − MX:LHPC (1:2.5), HSD − MX:LHPC (1:0), HSD − MX:LHPC (1:1), HSD − MX:LHPC (1:2.5), and HSD − MX:LHPC (1:5).

Figure 8 shows the MX release from hydrogels at an HPG concentration of 1.75% (*w*/*v*) containing: MX raw material (HMX − RM), physical mixture (HPM − MX:LHPC (1:2.5)) and the different MX-SD solid dispersions HSD-M:LHPC (1:0), HSD-M:LHPC (1:2.5) and HSD-M:LHPC (1:5) in acetate buffer of pH 5.8 and at 32 ± 1 °C (simulated skin medium). The concentration of this gelling agent (HPG) generated a hydrogel with viscosity values suitable for topical administration [42].

The dissolution rate profile of HMX − RM hydrogel showed an initial slow release at 30 min of 16.60 ± 0.78% and a sustained release rate up to 2 h (20.43 ± 0.76%). Similar slow dissolution profiles for poorly soluble drugs, such as MX, have been previously reported [21]. The delayed dissolution profile of hydrogels containing 1.75% HPG was related to its viscosity [6,37].

The physical mixture hydrogel HPM − MX: LHPC (1:2.5) exhibited a slight increase for the initial release at 30 min (20.30 ± 0.81%) and showed a significant increase in drug release rate (*p* < 0.05) at 2 h (32.22 ± 0.93%) compared to HMX − RM. The existence of hydrophilic swellable additives such as LHPC enhances drug wetting and diminishes the interfacial tension between MX and the dissolution solution, thus resulting in a relatively increased dissolution rate [14,21].

The solid dispersion hydrogel HSD − MX:LHPC (1:0) showed a slight improvement in its release profile with respect to HMX − RM, in dissolution percentages at 2 h (1.72-fold). Possibly, the inclusion of SDS in HSD − MX:LHPC (1:0) along with the freeze-drying process might enhance drug wetting and solubilization, thereby enhancing the dissolution rate of MX [14]. However, the absence of LHPC in this formulation, caused a slightly lower release profile than HPM − MX:LHPC (1:2.5) hydrogel. This result indicated that the presence of the hydrophilic carrier LHPC resulted in considerable improvements in the wettability and solubility of poorly soluble drug [29].

The solid dispersion hydrogels HSD − MX:LHPC (1:1) and HSD − MX:LHPC (1:2.5) demonstrated a notable increase (*p* < 0.05) in comparison to HMX − RM in the dissolution studies. The FTIR and DSC studies for these solid dispersions indicated the presence of the amorphous form of MX, thus improving their solubility at pH 5.8. Moreover, drug release in both hydrogels HSD − MX:LHPC (1:1) and HSD − MX:LHPC (1:2.5), exhibited high increments at 30 min (3.13 and 3.49-fold) and at 2 h (3.20 and 3.97 -fold), respectively, compared to HMX − RM hydrogel. This could be due to an important decrease in crystallinity observed for the solid dispersions studied in the DSC and XRPD studies. Furthermore, the presence of hydrophilic chains of LHPC within the network favored the wettability of the hydrophobic MX particle’s surface and improved their solubility and dissolution profile [14,43].

Finally, the dissolution results of HSD − MX:LHPC (1:5) did not show significant improvements compared to HSD − MX:LHPC (1:2.5). This result indicates that the solubility and the slight improvement in the reduction of crystallinity observed in the DSC studies for the SD − MX:LHPC (1:5) does not achieve significant improvements in the MX transfer studies compared to the HSD − MX:LHPC (1:2.5). Possibly, the greater number of LHPC chains within the interpolymeric network did not allow the improvement of the MX release rate [29].

#### 2.5.5. Kinetic Studies

Drug release kinetics for hydrogels containing: MX raw material (HMX − RM), physical mixture (HPM − MX:LHPC 1:2.5), and the solid dispersions HSD − MX:LHPC (1:0), HSD − MX:LHPC (1:1), HSD − MX:LHPC (1:2.5), and HSD − MX:LHPC (1:5) are shown in Table 1 and Table 2.

The kinetic study of the HMX-MR showed good fitting results to both zero-order and first-order models (*r*^2^ of 0.9952 and 0.9885, respectively). The low values of *K*_0_ (0.0011 min^−1^) and *K*_1_ (−0.0013 min^−1^) for HMX − RM were related to slow drug diffusion. The *n* value for the Korsmeyer–Peppas model (0.9862) was related to a combination of erosion and diffusion mechanisms (see Table 1 and Table 2). The poor solubility of MX in HPG hydrogel has been previously observed [6,34]. However, the zero-order model shows the best fit for the release kinetic data of both hydrogels HPM − MX:LHPC (1:2.5) and HSD − MX:LHPC (1:0) (*r*^2^ 0.9975 and 0.9983, respectively), and both of them exhibited a significant increase for values of *K*_0_ (0.0020 and 0.0019 min^−1^) compared to HMX − RM. The Korsmeyer–Peppas model for both hydrogels HPM − MX:LHPC (1:2.5) and HSD − MX:LHPC (1:0) presented high *n* values of 0.9798 and 0.905, respectively, which were related to an anomalous non-Fickian kinetics which combines erosion and diffusion mechanisms. The wettability observed for these systems was attributed to the hydrophilic effect of LHPC and the freeze-drying process. Comparable fitting effects were observed in systems with other hydrophilic carriers with the Korsmeyer–Peppas model [34,43].

The three solid dispersion hydrogels HSD − MX:LHPC (1:1), HSD − MX:LHPC (1:2.5) and HSD − MX:LHPC (1:5) produced a spreading of the MX molecules within the LHPC chains. Both HSD − MX:LHPC (1:1) and HSD − MX:LHPC (1:2.5) hydrogels exhibited a good fit to the first-order model (*r*^2^ 0.9992 and 0.9852, respectively), with important improvements in the values of *K*_1_ (−0.0080 and −0.0160 min^−1^, respectively) compared to HMX − RM. For the Korsmeyer–Peppas model, both systems showed *n* values of 1.0516 and 1.1674, respectively (see Table 2), indicating that both hydrogels followed the Case-II release mechanism. The elevation in the *n* values in comparison to HMX − RM has been associated with polymer relaxation and rapid drug dissolution. Similar kinetic parameters have been previously observed [34,43]. The HSD − MX:LHPC (1:5) hydrogel presented a better fit to the first-order model (*r*^2^ 0.9964). The value of *K*_1_ (−0.0125 min^−1^) for this hydrogel was lower compared to HSD − MX:LHPC (1:2.5) (see Table 2), while its value of *n* (1.1583), suggesting an elevated polymer relaxation, characteristic of Case-II release mechanism for Korsmeyer–Peppas model. These results indicated that a higher LHPC ratio in HSD − MX:LHPC (1:5) hydrogel does not really improve the MX release from HPG hydrogels [29].

## 3. Conclusions

A process of elaboration and characterization of different hydroxypropyl guar hydrogels based on solid dispersions of diverse MX:LHPC ratios: 1:1; 1:2.5; 1:5; 1:10 were carried out. The decline in drug crystallinity and the increase in the presence of hydrogen bonds for the different solid dispersions have been related to improvements in MX dissolution from HPG hydrogels. Formulating solid dispersions with low LHPC ratios as a carrier, such as SD − MX:LHPC (1:1) and (1:2.5), leads to significant enhancements in solubility (4.61 and 5.64-fold, respectively).

DSC studies of SD − MX:LHPC (1:1) and SD − MX:LHPC (1:2.5) showed that increases in LHPC ratios were related to significant decreases in MX crystallinity percentages (18.12 and 8.62%, respectively). These crystallinity decreases were confirmed by XRPD studies. However, the DSC studies indicated that the higher LHPC ratios in SD − MX:LHPC (1:5) did not achieve improved crystallinity reduction compared to SD − MX:LHPC (1:2.5). The FTIR showed hydrogen bonds characteristics of drug/polymer and polymer/polymer interactions for the different solid dispersions.

HPG hydrogels at a concentration of 1.75% (*w*/*v*) with different solid dispersions were developed to improve the topical release of MX. Viscosity, SEM, and FTIR studies showed changes in the polymer/polymer interactions and in the surface morphology of the HSD − MX:LHPC (1:2.5) hydrogel. These changes were related to a high increase in the hydrogen bonds among hydroxyl groups from the LHPC and HPG polymeric chains with the water molecules within the hydrogel network. In vitro drug release from MX hydrogels (HMX − RM) showed a slow drug release characteristic of poorly soluble drugs. The presence of hydrophilic swellable excipients as LHPC in HPM − MX:LHPC (1:2.5) improves the wettability and enhances the MX release profile.

The slow drug release profiles for HMX − RM, HPM − MX:LHPC (1:2.5), and HSD − MX:LHPC (1:0) showed a good fit to zero-order and Korsmeyer–Peppas. The hydrogels based on solid dispersions HSD − MX:LHPC (1:1) and HSD − MX:LHPC (1:2.5) displayed high increases in drug release profiles with increases of 3.20 and 3.97-fold at 2 h compared to HMX − RM, respectively. The first-order and Korsmeyer–Peppas models provided the most suitable fit for HSD − MX:LHPC (1:1) and (1:2.5) because of their rapid release kinetics. Possibly, the intramolecular hydrogen bonds for the LHPC and HPG polymeric chains promote hydration, increase the mobility of the drug in its amorphous form, and improve the MX release.

## 4. Materials and Methods

### 4.1. Substances and Reagents

Meloxicam (MX) employed in the research was supplied by Normon (Madrid, Spain). Sodium dodecyl sulfate (SDS) was provided by Fischer Scientific, Loughborough, UK. Low-substituted hydroxypropyl cellulose (LHPC, with a hydroxypropoxy content ranging from 5% to 16%, and a molecular weight between 30,000 to 150,000) was supplied by Shin-Etsu^®^, Tokyo, Japan. Hydroxypropyl guar (HPG, molecular weight of 90 KDa, with a substitution level of 1.2) was purchased from Seppic^®^ (Barcelona, Spain). The water utilized in these experiments was sourced from a Milli-Q water purification system (Billerica, MA, USA). All remaining chemicals met or exceeded pharmaceutical-grade standards.

### 4.2. Preparation of Solid Dispersions (SDs)

MX solid dispersions were formulated via the freeze-drying technique employing LHPC as a carrier. SDs were developed utilizing the subsequent ratios of MX:LHPC: 1:0; 1:1; 1:2.5, 1:5 and 1:10 (*w*/*w*). For each formulation, 20 mg of MX was dissolved by stirring in 20 mL of alkalized solution at pH 8.8 with 0.2 M sodium hydroxide, using 0.125 mg/mL of SDS as a humectant. Then the required quantities of LHPC were weighed and co-dispersed. The samples were subsequently frozen at –40 °C for 24 h and subjected to freeze-drying using a Liolabar^®^ 7 (Telstat Inc., Madrid, Spain). Following the freeze-drying process, each formulation was ground and sieved to achieve a particle size range of 0.125–0.500 mm. The vials were then stored at room temperature in a desiccator filled with silica gel. The physical mixture (PM) of MX:LHPC in a ratio of 1:2.5 (*w*/*w*) was formulated by manually mixing the appropriate amount of MX with particle size fractions of 0.125–0.500 mm, SDS, and carrier in a ceramic vessel using a polymeric spatula.

### 4.3. Preparation of Hydrogel Formulations

Firstly, a study was carried out with control hydrogels (H-blank) with different percentages of HPG (1.0–2.5% (*w*/*v*)) to evaluate their viscosity characteristics for topical administration. To prepare the control hydrogel (H-Blank), the different amounts of HPG polymer were dispersed in a pH 5.8 phosphate buffer by stirring at 600 rpm, to obtain a homogeneous dispersion. The hydrogel was allowed to stand so that any entrained air could escape. Then, to 5 mL of the hydrogel already formed, it was added quantities of MX, PM − MX:LHPC (1:2.5), or SD − MX:LHPC (1:2.5) equivalent to 65 mg of MX by stirring at 600 rpm for 2 min. These hydrogels will be used for viscosity and in vitro release rate studies.

A sample of the hydrogels at an HPG concentration of 1.75% (*w*/*v*): H-Blank, HPM − MX:LHPC (1:2.5), and HSD − MX:LHPC (1:2.5) underwent freeze-drying for scanning electron microscopy (SEM) and Fourier-transform infrared spectroscopy (FTIR) analyses.

### 4.4. Solubility Study

Solubility tests were conducted for the following samples: MX raw material (MX − RM), physical mixture (PM − MX:LHPC 1:2.5), and the different solid dispersions (MX:LHPC − 1:0; MX:LHPC − 1:1; MX:LHPC − 1:2.5; MX:LHPC − 1:5, and MX:LHPC − 1:10). For solubility studies, 5 mg of MX or its equivalent amount were weighed and added to 5 mL of pH 5.8 buffer solution in a temperature-controlled bath at 32 ± 1 °C, agitating for a duration of 3 days. The samples underwent filtration and dilution with a pH 5.8 buffer for quantification. The total quantity of MX released from the formulations was assessed at 364 nm utilizing a UV-VIS JASCO^®^ V-730 spectrophotometer (Jasco International Co., Ltd.; Tokyo, Japan). The evaluation was conducted using the provided calibration curve: y = 0.0074x (µg/mL) + 0.0101 (r^2^ = 0.9997), covering a concentration range of 2–15 µg/mL. Each measurement at every time point was conducted three times, and the error bars displayed on the graphs indicate the standard deviation.

### 4.5. Differential Scanning Calorimetry (DSC)

DSC (Differential Scanning Calorimetry) analyses for the various formulations were conducted utilizing an automated thermal analysis system (Mettler^®^ Toledo TC 15, TA controller, Schwerzenbach, Switzerland). Temperature calibration was carried out using the Indium Calibration Reference Standard (transition point 156.60 °C). Each dried sample was precisely weighed into aluminum pans, sealed hermetically with aluminum lids, and then subjected to heating from 25 to 320 °C at a rate of 10 °C/min under a constant flow of dry nitrogen at 30 mL/min. An empty pan, subjected to the same conditions, was utilized as a reference. The absolute percent crystallinity, X (%), was calculated from

(1) $$X\left(\%\right)=(∆HMX-SD/∆HMX-RM),$$

where ΔHMX − SD is the enthalpy of fusion of MX for different solid dispersions. This is calculated as the quotient of the enthalpy of fusion of the sample (MX-SD) divided by the composition of MX, and ΔHMX − RM represents the enthalpy of fusion of 100% crystalline MX − RM at the same heat rate [26].

### 4.6. X-ray Powder Diffractometry (XRPD)

The XRPD analyses of the various formulations MX and LHPC raw material (MX − RM), LHPC, physical mixture PM − MX:LHPC (1:2.5) and solid dispersions: SD − MX: LHPC (1:0); SD − MX:LHPC (1:1); SD − MX:LHPC (1:2.5) and SD − MX:LHPC (1:5) were conducted using a Philips^®^ X’Pert-MPD X-ray diffractometer (Malvern Panalytical; Almelo, The Netherlands) at the CAI (Centro de Asistencia a la Investigación, Complutense University of Madrid, Madrid, Spain). The samples were irradiated with monochromatic CuKα radiation (λ = 1.542 Å) and scanned over the 5–50 (2θ) range with a step size of 0.04 and a dwell time of 1 s per step. A voltage of 30 kV and a current of 30 mA were applied.

### 4.7. Fourier-Transform Infrared Spectroscopy (FTIR)

The various samples were prepared by weighing different quantities of the respective formulations (equivalent to 2 mg of MX) and blending them with 100 mg of potassium bromide. Subsequently, the samples underwent compression at 10 T using a Carver hydraulic press Model C-3912 (Wabash, IN, USA). Fourier-transform infrared spectroscopy (FTIR) analysis was conducted using a Perkin Elmer^®^ 1600 FTIR spectrophotometer (Perkin Elmer, Inc., Hopkinton, MA, USA). The spectra were acquired at a resolution of 2 cm^−1^, averaging 16 scans with a scanning speed of 2 mm per second. The infrared region was examined within the range of 400–4000 cm^−1^.

### 4.8. Viscosity Study

Viscosities of HPG hydrogels at a concentration of 1.75% (*w*/*v*) were measured by using a Brookfield rheometer, model DV-III (Middleborough, MA, USA) equipped with a temperature control probe, utilizing a 200 μm gap and plate/plate configuration with a 40 mm diameter. The temperature for all measurements was maintained at 25 ± 0.5 °C. A rheological test was conducted to assess the flow characteristics of the following hydrogels: HPG blank hydrogel (H-Blank), MX raw material hydrogel (HMX − RM), physical mixture hydrogel HPM − MX: LHPC (1:2.5) and solid dispersion hydrogel HSD- MX: LHPC (1:2.5). Every sample was subjected to 55 measurements with a speed ramp from 0.5 to 700 s^−1^, each lasting 5 s. Three replicates were performed for each formulation, and viscosity curves at shear rates were plotted to understand the flow properties. The average viscosity measurement (Pa·s) was determined from the uniform shear section at 50 s^−1^ for each formulation.

### 4.9. Scanning Electron Microscopy (SEM)

Samples were affixed onto a double-sided adhesive tape and coated with a thin layer of gold–palladium using an Emitech K550X sputter coater (Quorum Technologies; Lewes, UK). Following coating, the freeze-dried hydrogel samples were examined using a Jeol^®^ JSM-6400 scanning electron microscope (Jeol Ltd., Peabody, MA, USA). All micrographs were generated through secondary electron imaging for surface morphology analysis, with an accelerating voltage set at 20 kV and a magnification of 500×.

### 4.10. In Vitro Drug Release

Drug release studies were performed with hydrogel composed of pure MX (HMX − RM), physical mixture hydrogel HPM − MX:LHPC (1:2.5) and solid dispersion hydrogels HSD − MX:LHPC (1:0); HSD − MX:LHPC (1:1); HSD − MX:LHPC (1:2.5); HSD − MX:LHPC (1:5), and HSD − MX:LHPC (1:10).

These studies were performed using the United States Pharmacopeia (USP) paddle over disk method (apparatus 5) in Erweka^®^ DT 80 (Erweka GmbH; Langen, Germany) dissolution equipment with a rotational speed of 50 rpm, at a temperature of 32.0 ± 0.5 °C, and 300 mL of pH 5.8 phosphate buffer. Each disk is filled with 0.25 mL of the different hydrogel formulations (all containing the equivalent of 3.2 mg of MX). A 5 mL sample was filtered (Acrodisc^®^ HPVL 0.45 µm, Port Washington, NY, USA) at 5,15, 30, 45, 60, 90, and 120 min. The cumulative MX release was spectrophotometrically analyzed at 360 nm by the method previously described in the solubility studies. Each determination at each time was performed in triplicate and the error bars on the graphs represent the standard deviation. Statistical analysis was used to compare the different results. A *p*-value of less than 0.05 was considered a criterion for a statistically significant difference (Statgraphics^®^ Plus, version 5.1).

### 4.11. Drug Release Kinetics

The release kinetics were fitted using zero-order release kinetics, the first-order kinetics model, and the Higuchi model [29].

(2) $$M_{t}/M_{\infty }=K_{0}\;t\;\mathrm{Zero}-\mathrm{order}\;\mathrm{model}$$

(3) $${Ln\;[100-(M}_{t}/M_{\infty })]=-K_{1}\;t\;\mathrm{First}-\mathrm{order}\;\mathrm{model}$$

(4) $$M_{t}/M_{\infty }=K_{H}\;t^{0.5}\;\mathrm{Higuchi}\;\mathrm{model}$$

The fractional drug released was described as M_t_ and the total amount released was represented as M_∞_. The kinetic dissolution constants for the zero-order, first-order, and Higuchi kinetic models were *K*_0_ (min^−1^), *K*_1_ (min^−1^) and *K_H_* (min^−1/2^), respectively, which characterize release as a function of time *t*.

Furthermore, the effects of drug/polymers interactions on the MX release were analyzed according to the Korsmeyer–Peppas Equation for M_t_/M_∞_ < 0.6, which can be expressed as the following Equation [29].

(5) $$M_{t}/M_{\infty }=k_{d}\;t^{n}\;\mathrm{Korsmeyer}–\mathrm{Peppas}\;\mathrm{Equation},$$

where M_t_/M_∞_ is the fractional drug released at time *t* (min), *Kd* (min^−n^) is the kinetic dissolution constant, and n is a diffusional exponent characteristic of the release as a function of time *t*. In the drug release, the exponent *n* is the diffusional constant that characterizes the drug release transport mechanism. When *n* = 0.43, a Fickian diffusion process was observed; the drug diffuses through the polymeric network, which is the dominant release mechanism. When the *n* values are 0.43 < *n* < 1, an anomalous transport (non-Fickian) drug diffusion occurs. Anomalous diffusion transport assumed that the mechanism of MX release was a combination of swelling, erosion, and diffusion. When *n* ≥ 1, a Case II transport could be observed. The *n* value ≥ 1.0 indicates that polymer relaxation, polymer dissolution, or erosion are the dominant mechanisms [29].

## Figures and Tables

**Table 1:** Zero-order, first-order, and Higuchi kinetic models applied for MX release from HMX − RM, the physical mixture hydrogel HPM − MX:LHPC (1:2.5), and the four solid dispersions hydrogels: HSD − MX:LHPC (1:0), HSD − MX:LHPC (1:1), HSD − MX:LHPC (1:2.5), and HSD − MX:LHPC (1:5) with corresponding release rate constant of zero-order *K*_0_ (min^−1^), first-order *K*_1_ (min^−1^), Higuchi model *K_H_* (min^−1/2^), and correlation coefficient (*r*^2^).

Formulations	Kinetic Models	*K*	*r* ^2^
H − MX − RM	Zero-order	0.1073	0.9952
	First-order	−0.0013	0.9981
	Higuchi model	0.0099	0.9935
H − PM − MX:LHPC (1:2.5)	Zero-order	0.2048	0.9975
	First-order	−0.0029	0.9934
	Higuchi model	2.0806	0.9820
H − SD − MX:LHPC (1:0)	Zero-order	0.1919	0.9983
	First-order	−0.0025	0.9973
	Higuchi model	2.5637	0.9950
H − SD − MX:LHPC (1:1)	Zero-order	0.2891	0.9814
	First-order	−0.0080	0.9992
	Higuchi model	6.0958	0.9873
H − SD − MX:LHPC (1:2.5)	Zero-order	1.1700	0.9945
	First-order	−0.0160	0.9852
	Higuchi model	6.7841	0.9877
H − SD − MX:LHPC (1:5)	Zero-order	0.486	0.9788
	First-order	−0.00125	0.9964
	Higuchi model	6.4260	0.9968

**Table 2:** Korsmeyer–Peppas kinetic model applied for MX release from HMX − RM, physical mixture hydrogel HPM − MX:LHPC (1:2.5), and the four solid dispersion hydrogels: HSD − MX:LHPC (1:0), HSD − MX:LHPC (1:1), HSD − MX:LHPC (1:2.5), and HSD − MX:LHPC (1:5) with corresponding diffusion exponent (*n*), release rate constant of Korsmeyer–Peppas *K_d_* (min^−n^) and correlation coefficient (*r*^2^).

Formulations	Korsmeyer–Peppas Kinetic Model		
	*n*	*K_d_* (min^−n^)	*r* ^2^
HMX − RM	0.9862	0.1611	0.9964
HPM − MX:LHPC (1:2.5)	0.9798	0.2669	0.9947
HSD − MX:LHPC (1:0)	0.2964	0.2964	0.9816
HSD − MX:LHPC (1:1)	0.4462	0.4462	0.9375
HSD − MX:LHPC (1:2.5)	0.3820	0.3820	0.8982
HSD − MX:LHPC (1:5)	0.3993	0.3993	0.9880

## Data Availability

The data presented in this study are openly available in article.
